# Host-Related Factors as Targetable Drivers of Immunotherapy Response in Non-Small Cell Lung Cancer Patients

**DOI:** 10.3389/fimmu.2022.914890

**Published:** 2022-07-06

**Authors:** Denisa Baci, Elona Cekani, Andrea Imperatori, Domenico Ribatti, Lorenzo Mortara

**Affiliations:** ^1^ Molecular Cardiology Laboratory, IRCCS-Policlinico San Donato, San Donato Milanese, Milan, Italy; ^2^ Immunology and General Pathology Laboratory, Department of Biotechnology and Life Sciences, University of Insubria, Varese, Italy; ^3^ Medical Oncology Clinic, Oncology Institute of Southern Switzerland, Bellinzona, Switzerland; ^4^ Center for Thoracic Surgery, Department of Medicine and Surgery, University of Insubria, Varese, Italy; ^5^ Department of Basic Medical Sciences, Neurosciences and Sensory Organs, University of Bari Aldo Moro Medical School, Bari, Italy

**Keywords:** non-small cell lung cancer, tumor microenvironment, immunotherapy, immune checkpoint inhibitors, anti-angiogenic therapies

## Abstract

Despite some significant therapeutic breakthroughs leading to immunotherapy, a high percentage of patients with non-small cell lung cancer (NSCLC) do not respond to treatment on relapse, thus experiencing poor prognosis and survival. The unsatisfying results could be related to the features of the tumor immune microenvironment and the dynamic interactions between a tumor and immune infiltrate. Host–tumor interactions strongly influence the course of disease and response to therapies. Thus, targeting host-associated factors by restoring their physiologic functions altered by the presence of a tumor represents a new therapeutic approach to control tumor development and progression. In NSCLC, the immunogenic tumor balance is shifted negatively toward immunosuppression due to the release of inhibitory factors as well as the presence of immunosuppressive cells. Among these cells, there are myeloid-derived suppressor cells, regulatory T cells that can generate a tumor-permissive milieu by reprogramming the cells of the hosts such as tumor-associated macrophages, tumor-associated neutrophils, natural killer cells, dendritic cells, and mast cells that acquire tumor-supporting phenotypes and functions. This review highlights the current knowledge of the involvement of host-related factors, including innate and adaptive immunity in orchestrating the tumor cell fate and the primary resistance mechanisms to immunotherapy in NSCLC. Finally, we discuss combinational therapeutic strategies targeting different aspects of the tumor immune microenvironment (TIME) to prime the host response. Further research dissecting the characteristics and dynamic interactions within the interface host–tumor is necessary to improve a patient fitness immune response and provide answers regarding the immunotherapy efficacy, with the aim to develop more successful treatments for NSCLC.

## Introduction

Lung cancer has become a leading cause of cancer death worldwide. The global incidence sees polarized differences according to the economic development of different countries. There is a decrease in the incidence among men in high-income countries due to public health measures and a gradual and progressive increase in both genders in low-income countries where public health initiatives for smoking cessation have lagged and access to healthcare is scarce (1–3). During the first decade of the present century, the outcomes of at least a subset of patients have seen substantial improvements, thanks to a general understanding of disease biology, the application of predictive biomarkers, and refinements in specific treatments (4).

Non-small cell lung cancer (NSCLC) represents approximately 85% of all lung cancer cases. It includes three major histologic classifications: adenocarcinoma (ADC), representing the most common subtype of lung cancer, followed by squamous-cell carcinoma (SCC) and large-cell carcinoma (LCC) (5).

Treatment is highly dependent on many parameters of the patient, particularly their general functional status, comorbidities, the tumor stage, and the molecular features of the disease. The primary treatment for stages I and II and in selected cases for stage III A disease is curative surgery, chemotherapy, radiation therapy (RT), or a combined modality approach. Postoperative adjuvant cisplatin-based chemotherapy is recommended in patients with completely resected stage II–IIIA disease and selected patients with stage IB disease (6). However, this therapy is associated with only a 16% decrease in the risk of disease recurrence or death; for 5 years, it is associated with a 5% decrease in the risk of death (7, 8).

Systemic therapy is pursued in the cases of patients with stage IV disease and in presence of metastases or in the presence of relapse after initial management.

Over a median follow-up of approximately five years, the percentage of patients who have disease recurrence or who die after surgery remains high (ranging from 45% among patients with stage IB disease to 76% among those with stage III disease), regardless of the use of postoperative chemotherapy (8). The opportunity for improving survival is pronounced in early-stage disease and is driving studies integrating targeted therapies and immune checkpoint inhibitors (ICIs). As a result, after the revolutionary data on the metastatic setting of epidermal growth factor receptor (EGFR) inhibitors (9–13), we, nowadays, have the impressive results of the Adaura trial, which led to an important improvement of disease-free survival (DFS) in a subset of patients with EGFR-mutated early-stage lung cancer when osimertinib was added as an adjuvant treatment to the main treatment for the duration of 3 years (14). Unfortunately, this subgroup is limited to only patients with EGFR-targetable mutations.

From the past decade to the present, with additional activating genomic alterations such as those affecting anaplastic lymphoma kinase (ALK), ROS1 proto-oncogene receptor tyrosine kinase, class 1 B-Raf proto-oncogene (BRAF) mutations (V600), mesenchymal-epithelial transition factor (MET), and neurotrophic receptor tyrosine kinase (NTRK) ALK, ROS1, B-Raf V600, MET, and NTRK alterations and the availability of an increasing number of specific tyrosine-kinase inhibitors (TKIs) of various generations, the proportion of patients with an improved prognosis has further increased (15–17).

The second pilar of the modern treatment of metastatic NSCLC is taken by immunotherapy (PD-1 and PD-L1 monoclonal antibodies), which is nowadays in the frontline of treatment in oncogenic driver–negative NSCLC and has produced response and survival rates that were unreachable a few years ago (18, 19).

Patients whose tumors express PD-L1 in at least 50% of the cells are more likely to attain a response and survive longer if treated with these compounds.

After breakthrough immune checkpoint inhibitor data in an advance setting, we now have the first results of immunotherapy in an adjuvant setting. The study IMpower 010 (20) addressed some of the unmet needs for adjuvant treatment oncodriver-negative tumors, adding immunotherapy in the plethora of new approvals in the early setting of NSCLC for patients expressing PD-L1 >1% on tumor cells (20).

In the meantime, immunotherapy continues to demonstrate a significant overall survival (OS) benefit in advanced NSCLC. In particular, pembrolizumab or atezolizumab monotherapies are superior to first-line chemotherapy in tumors with a higher expression of the PD-L1 molecule (21, 22). Interestingly, different chemo-immunotherapy combinations have been shown to be superior to chemotherapy, regardless of PD-L1 expression (23, 24).

Even though immunotherapy can produce great and long-lasting results, not all NSCLC patients seem to benefit from this approach (25). Many attempts have been made to identify predictive biomarkers to select responding patients who would benefit from ICIs. Tumor mutational burden (TMB) is a critical predictive factor for response to immunotherapy, but the available results need further confirmation in prospective randomized trials (26). A critical factor underlying the poor response to immunotherapies is the heterogeneity in the immune cell response to NSCLC and the existence of multiple mechanisms mediating tumor immune suppression (27). Indeed, a limited knowledge of the characteristics of the TME, to a great extent, hinders the development of new targets for immunotherapy. Here, we review the biological functions of immune cells within the tumor immune microenvironment (TIME) and their roles in cancer immunotherapy and discuss the perspectives of the basic and translational studies for improving the effectiveness of the clinical use.

## Overview of NSCLC Tumor Immune Microenvironment

**Figure 1:**
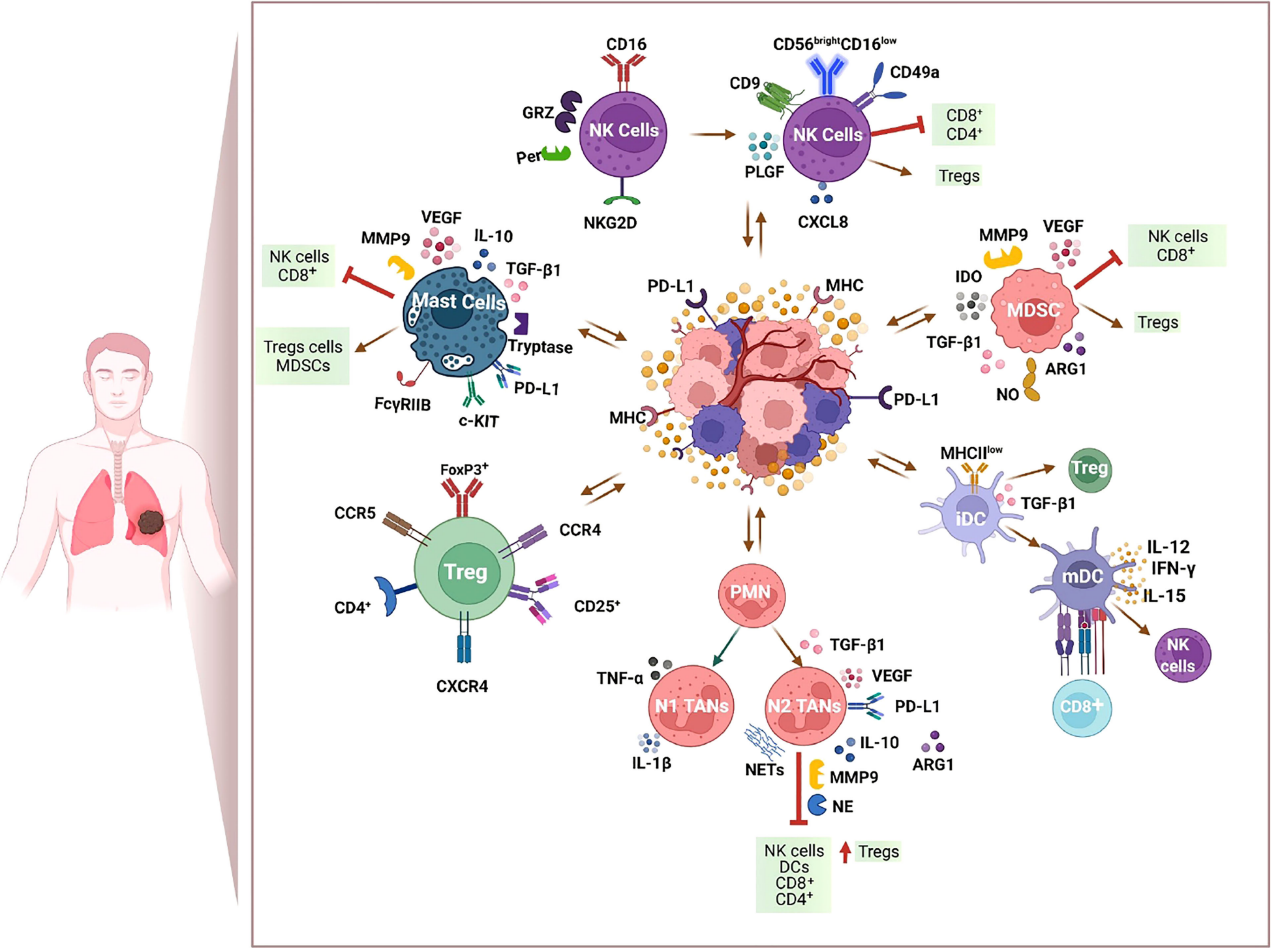
Immunosuppressive milieu within the tumor immune microenvironment (TIME) of NSCLC. Immunotherapy resistance is marked by an immunosuppressive TIME and includes tumor-derived factors, infiltration of T regulatory cells (Tregs), myeloid-derived suppressor cells (MDSCs), and mast cells that, in turn, favor the polarization toward a protumor phenotype of other immune cells such as neutrophils, dendritic cells (DCs), and natural killer (NK) cells. Figure created with http://biorender.com.

Taking advantage of new technologies (e.g., single-cell RNA sequencing), multiple ongoing studies are now identifying new subtypes of tumor-associated immune cells to predict the clinical efficacy of different immunotherapy approaches. The study of immune tumor cell contexture in cancer patients to target the multiple immune-suppressive factors might ameliorate response rates and contribute to develop the era of personalized immune-based therapies. The immune cell populations present in the TIME possess both tumor-killing potentials and may alternatively promote or suppress immune cell activity. Tolerogenic immune cell populations such as regulatory T cells (Tregs) and myeloid-derived suppressor cells (MDSCs) create an immune-suppressive milieu that, in turn, favors the polarization toward a protumor phenotype of other immune cells such as neutrophils, dendritic cells (DCs), and natural killer (NK) cells (**Figure 1** and **Table 1**). Understanding these immunologic states and the mechanisms underpinning them may provide the key to restore an effective anti-tumor immune response and improve the survival rate of NSCLC patients.

**Table 1:** NSCLC immune landscape: anti- and pro-tumorigenic phenotypes and activities of immune cell populations within the tumor microenvironment.

Tumor immune microenvironment (TIME)	Cell subpopulations	Cell markers	Anti-tumor properties	Pro-tumor properties
Neutrophils	N1 TANs	CD11b^+^, CD66b^+^, CD15^+^, CD16^+^, HLA-DR^−^, TNF-α^high^, CXCR2^low^, CXCL8^low^	IFN-γ, IL1 and TNF-α-mediated stimulation of immune response; ROS-mediated tumor killing; Promote CD4^+^ T cell responses	NA
		N2 TANs	CD11b^+^, CD66b^+^, CD15^+^, CD16^+^, HLA-DR^−^, TNF-α^low^, CXCR2^high^, CXCL8^high^, ARG1^high^	NA	MMP9, NE, VEGF-mediated tumor metastasis and invasion; IL10, TGF-β, ARG1, NETs-mediated immune suppression; Suppression of NK cells and CD8^+^ T cells immune response
MDSCs	M-MDSCs	CD11b^+^, CD15^−^, CD14^+^, HLA-DR^−/low^, S100A9^+^, CD33^+^, ILT3^high^	NA	MMPs, VEGF -mediated angiogenesis, invasiveness and metastasis; IL10, TGF-β, IDO, ARG1, and PGE-mediated immunosuppression; Suppression of NK cells, DCs the functions; Suppression of CD8^+^ T cells antitumor response; Tregs differentiation and expansion
	PMN-MDSCs	CD11b^+^, CD14^−^, CD15^+^, CD66b^+^, HLA-DR^−/low^, Lox-1^+^		
NK cells	Cytotoxic NK cells	CD56^dim^, CD16^+^, Perf^high^, GRZ^high^, TNF-α^high^, IFN-γ^high^, NKG2D^high^	Cytotoxic-mediated apoptosis of cancer cells; DCs maturation by releasing IFN-γ;	NA
	Immature/decidua-like NK Cells	CD56^bright^, CD16^low/−^, Perf ^low^, IFN-γ ^low^, TNF-α^low^ NKG2A^high^, NKG2D^low^, CTLA-4^+^, PD-1^+^, CD9^+^, CD49a^+^, CXCL8^+^	NA	Anergic NK cells-mediated tumor immune evasion; Angiogenesis induction releasing VEGF, PlGF, CXCL8; Suppression of DCs and CD8^+^ T cells functions
NKT cells	Type I NKT	TCR binding with a α-GalCer, CD3^+^, CD4^+^, CD8^+^, CD56^+^, CD161^+^	Killing of CD1d^+^ tumor cells; IFN-γ-mediated stimulation of CD8^+^ T cells immune response; Activation of NK cells	NA
	Type II NKT	TCR bindings with sulfatide-loaded CD1d, CD3^+^	IFN-γ-mediated suppression of tumor growth	IL13-mediated immunosuppression
DCs	mDCs	HLA-DR^+^, CD80^+^, CD83^+^, CD86^+^, CD208/DC-LAMP^+^	Th1 cytotoxic immune response; Stimulation of CD8^+^ T cells immune response;	NA
	iDCs	HLA-DR^low^, CD80^low^, CD83^low^, CD86^low^, CD208/DC-LAMP^low^	Antigen presentation to T cells	Immunosuppression
Tregs	CD3^+^, CD4^+^, CD25^+^, FoxP3^+^, CTLA4^+^, CD127^low^, PD-1^+^, CTLA-4^+^, CD39^+^, CD73^+^		NA	Suppression of CD8^+^ T cells mediated immune response
Mast cells	FcϵR1α^+^, FcγRIIb/CD32^+^, CD117/c-kit^high^, CD203c^+^, Tryptase^+^, CD103^+^		Promote CD4^+^ T cell responses	MMPs, chymase and tryptase-mediated metastasis; VEGFA-mediated angiogenesis

ARG1, arginase-1; HLA, human leukocyte antigen; iDCs, immature dendritic cells; IDO, indoleamine 2,3-dioxygenase; LOX-1, Lectin-like oxidized low-density lipoprotein (LDL) receptor-1; mDCs, mature dendritic cells; MDSCs, Myeloid-derived suppressor cells; MMP-9, matrix metalloproteinase-9; NA, not applicable; NETs, neutrophil extracellular traps; NK, natural killer; NKT, natural killer T; PMN-MDSCs, Polymorphonuclear-MDSCs; TANs, tumor-associated neutrophils; TGF-β, Transforming growth factor-beta; TIME, Tumor immune microenvironment; TNF-α, tumor necrosis factor-alpha; Treg, Regulatory T; VEGF, vascular endothelial growth factor; α-GalCer, glycolipid α-galactosylceramide.

### Neutrophils

Neutrophils are considered the first line of innate immune defense and are recognized as a critical targetable cellular feature of NSCLC TIME (28). Several preclinical and clinical studies have linked neutrophil trafficking and degranulation with various stages of tumor progression and the attenuation of treatment efficacy (29, 30). A high neutrophil/lymphocyte ratio (NLR) is now considered as a useful predictor associated with a negative clinical outcome, as well as with poor responsiveness to PD-1-/PD-L1 inhibitors (30). In accordance, Gentles et al. discovered that the neutrophil transcript signature was the strongest predictor of mortality and major infiltrating immune cells in adenocarcinoma NSCLC patients (31). In attempts to provide a clear description of the immune cell types present in NSCLC, Kargl et al. implicated neutrophils as the most abundant and dominant immune-suppressive factors associated with the depletion of CD4^+^ and CD8^+^ T lymphocytes within TIME (32). Consistent with previous findings, another study demonstrated a positive correlation between an increased tumor burden, high levels of neutrophil-related cytokines, and a dampened T-cell response associated with reduced CD3^+^CD8^+^ T-cell infiltration (33). However, the recruitment of neutrophils to the tumor microenvironment might depend on NSCLC subtypes and the smoking status, and larger studies are needed to define their role and association with survival (34).

Neutrophils in cancer consist of multiple heterogeneous cell populations and retain plasticity. A high expression of lectin-type oxidized LDL receptor 1 (LOX-1) distinguishes PMN-MDSCs from neutrophils (**Table 1**) (35). LOX-1^+^ PMN-MDSC numbers increased with anti-PD-1 therapy in non-responders, suggesting immunosuppressive functions in patients with NSCLC (36). A poor NSCLC prognosis and recurrence after surgery have been associated with increased circulating CD15^+^ LOX-1^+^ PMN-MDSCs, thus displaying potential as a diagnostic marker for NSCLC. In patients with advanced stages of lung cancer, there have been reports of the accumulation of low-density neutrophils (LDNs), CD66b^+^ PMNs, a subset of circulating neutrophils based on their sedimentation properties (37). Following tumor tissue infiltration and under specific tumor microenvironment cues, TANs can acquire a tumor- suppressive (N1) phenotype or become tumor-promoting/tolerogenic (N2) (**Figure 1** and **Table 1**). At the early stages of tumor development, N1 TANs predominate. A cytotoxic action of TANs and tumor regression was reported in a recent work employing a patient-derived xenograft (PDX) mouse model of early-stage NSCLC that received anti-PD-1 ICI, as a monotherapy or with cisplatin (38).

However, tumor growth, together with a shifted balance between IFN-β and TGF-β, can favor N2 neutrophils and/or PMN-MDSC accumulation. The release of tumor recruitment–soluble factors, such as CXCL8, CXCL1, CXCL5, CXCL7, IL-6, and IL-1β, enhances immunosuppressive neutrophil chemotaxis through CXCR2 sensing, found to be highly expressed in NSCLC patients (39, 40). In a murine lung cancer model, CXCR1/2 neutrophil receptor inhibition granted access to CD8^+^ T cells to the malignant tumor. Notably, the IFN-γ signature was restored, thus overcoming neutrophil-mediated immunosuppression and an associated mitigation of the effectiveness of PD-1-targeted immunotherapy (41). N2 TANS enhances the immunosuppressive milieu by expressing high levels of PD-L1, arginase-1 (ARG1), reactive oxygen species (ROS), nitric oxide (NO), IL-10, and TGF-β1, shaping the tumor landscape and impairing T-cell-mediated cytotoxicity (42) The expression of both CXCL8 and Arg-1 by neutrophils is correlated with ICI therapy failure and poor prognosis in NSCLC (43, 44). N2 TANs also increase angiogenesis by releasing pro-angiogenic factors such as vascular endothelial growth factor (VEGF), enhance extracellular matrix (ECM) remodeling, and foster a pre-metastatic niche formation by directly acting as the primary source of proteolytic enzymes (45, 46). High levels of an MMP9:tissue inhibitor of metalloproteinase-3 (TIMP-3) ratio have been found significantly elevated in NSCLC biopsies. Furthermore, neutrophil elastase (NE) and myeloperoxidase (MPO) high degranulation induce the formation of neutrophil extracellular traps (NETs), directly implicated in metastasis (47, 48) (**Figure 1**). Accordingly, NETs are involved in a vascular endothelium injury mediated by an inflammatory response (48), as well as the wrapping and shielding of tumor cells from cytotoxicity mediated by CD8^+^ T cells and NK cells (47, 49). The Inhibition of NETosis sensitizes tumors to PD-1 plus CTLA-4 inhibition (47).

Several therapeutic strategies to suppress N2 tumor-promoting phenotypes or reactivate their cytotoxic features toward cancer cells are in preclinical and clinical phases of evaluation (28). Main neutrophil- targeting approaches neutralize tumor-derived chemokines, promoting their influx within the tumor microenvironment and conversion to an MDSC-like phenotype/N2 TANS. Two recent studies suggested that targeting MDSCs *via* the antagonism of GM-CSF and fatty acid transport protein 2 (FATP2) by using lipofermata decreased ROS and PGE2-levels and their immunosuppressive functions in tumor-bearing mice (50, 51). Importantly, FATP2 enhanced anti-PD-L1 tumor immunotherapy and inhibited tumor progression (50, 51). Metformin also targets FATP2, disabling the suppressive capacity of granulocytic myeloid-derived suppressor cells eliciting Th1 and cytotoxic T lymphocytes (CTLs) responses (52). Retrospective studies suggest the synergistic actions of metformin and conventional chemotherapy, improving the survival and outcomes of patients with NSCLC (53, 54). Targeting key immunosuppressive factors in TIME such as TGF-β1 and chemokine receptors CXCR1/2 through pharmacological antagonists represent some of the strategies to block the immunosuppressive milieu, leading to tumor growth and the nonsuccess of immunotherapies.

### Myeloid-Derived Suppressor Cells

Myeloid-derived suppressor cells (MDSCs) represent a group of heterogeneous cells derived from immature myeloid progenitors with strong immunosuppressive features and functions. According to their phenotypic and morphological features, MDSCs have been classified into two major subsets: monocytic MDSCs (M-MDSCs) expressing CD14 and granulocytic or polymorphonuclear (PMN-MDSCs) expressing CD15 and CD66b; both types express CD33 in addition to CD11b with the absence of HLA-DR (**Table 1**). Even though PMN-MDSCs and neutrophils share similar phenotypic cell surface markers in humans, they have a distinct unique transcriptomic/phenotypic profile and functions that reflect the different roles within the tumor setting: PMN-MDSC but not neutrophils display immunosuppressive activities (55–57). A specific expression of LOX-1, fewer granules, and a reduced expression of CD16 can distinguish PMN-MDSCs phenotypically from neutrophils (**Table 1**) (35, 57, 58). In addition, neutrophils are high-density cells, whereas PMN-MDSCs are enriched in a low-density mononuclear cell fraction (55, 59). On the other hand, M-MDSCs can be distinguished from monocytes by detecting MHC class II, expressed only on monocytes (HLA-DR^+^) (60).

M-MDSCs and PMN-MDSCs share similar features; both enable immune response suppression but use different immunosuppressive mechanisms. For instance, PMN-MDSCs express high levels of ROS and low levels of NO, whereas M-MDSCs are the opposite. M-MDSCs preferentially exert their immunosuppressive functions by releasing IL-10, TGF-β, iNOS, and Arg-1 (56, 61).

Several studies have reported an accumulation of M-MDSCs in NSCLC patients (62–64). An increased pool of CD11b^+^CD14^⁻^CD15^+^CD33^+^ MDSCs and decreased CD8^+^ T cytotoxic lymphocytes have been reported in the peripheral blood of NSCLC patients (65). Another study reported a correlation between a subset of MDSC CD14^+^S100A9^+^, T-cell suppression mediated by arginase, iNOS, IL-13/IL-4Rα axis, and poor response to chemotherapy (66). Goeje et al. described increased levels of MDSCs expressing immunoglobulin-like transcript 3 (ILT3), identified as CD11b^+^CD14^−^ CD33^+^CD15^+^ HLA-DR^−^ILT3^high^, associated with the immunosuppressive function of ILT3 on DCs and with reduced survival (67).

MDSCs are recruited to the tumor site *via* chemokines such as CCL2 and CXCL8 (68, 69). Of notice, CXCL8 is linked to the recruitment and activation of MDSCs and neutrophils. Indeed, the serum levels of CXCL8 may predict the responses to immunotherapies (68, 70, 71). TGF-β signaling has also been reported to promote the recruitment of MDSCs into tumors (72) and directly induce the generation of CD39^+^CD73^+^ myeloid cells in NSCLC patients *via* the activation of mTOR-HIF-1 signaling (73, 74) (**Figure 1**). CD39^+^CD73^+^ MDSCs are a distinct immunosuppressive subset, and their frequency in NSCLC patients may be sufficient to predict the chemotherapeutic response (74).

MDSCs are the largest producer of indoleamine 2,3-dioxygenase (IDO), directly acting on the immunosuppressive pathway of anti-tumor CD8^+^ T lymphocytes and the increase of Treg cell activity in the lung tumor microenvironment (75) (**Figure 1**). In a preclinical model of lung cancer, it was demonstrated that MDSC-associated IDO modulates the *in vivo* and *ex vivo* differentiation of B regulatory cells (Bregs), an IL-10 producing subset of B cells, found to be reduced in tumor-bearing IDO deficient mice (IDO^-/-^) (76). The anti-immune functions of MDSCs involve different mechanisms such as the production of NO, ROS, and the elimination of arginine required for T lymphocyte functions. In a Kras^G12D^ GEMM of a lung adenocarcinoma model, the suppression of MDSC arginase activity by an ARG1 inhibitor restored T-cell function by increasing arginine (77). MDSCs could also enhance angiogenesis and metastasis through the production of MMP9 and VEGF (**Figure 1**). A tight association of PMN-MDSC number with a patient response to the ICI anti-PD-1 has been reported (36). An enhanced APC activity and increased frequency of CD8^+^ T or NK intracytoplasmic expression of IFN-γ, perforin, and granzyme were found following MDSC depletion (36). Accordingly, another study demonstrated an increased number and function of NK- and T-cell effectors in the tumor and enhanced therapeutic vaccination responses after the depletion of MDSCs (78). Therefore, the inhibition of MDSC functions represents the key therapeutic solution to restore anti-tumor T lymphocyte effector responses and successful immunotherapy. Until today, only a few preparations endorsed by the U.S. Food and Drug Administration (FDA) have been described to have prominent effects on the recruitment and function of MDSCs (e.g., ATRA, vitamin D, gemcitabine, and bevacizumab). Considering the promising results of targeting MDSCs in murine models of lung cancer, various clinical trials are now ongoing in NSCLC patients (NCT02922764; NCT03846310; NCT03801304; NCT04262388). Breakthroughs in this research area should promote the rational design of new strategies to target MDSCs to improve clinical responses to current immunotherapies.

### Natural Killer Cells

NK cells represent innate effector lymphocytes with abilities to counteract or limit both tumor cells and virus-infected cells (79). In humans, the cell surface expression of the CD56 marker is the main phenotype marker for NK cells in association with a negative lineage-defining signature (CD3^−^, CD14^−^, CD19^−^, and TCR^−^), whereas in mice, it is the NK1.1 marker. NK cells do not need specific antigen stimulation but are activated toward neoplastic or stressed cells through the fine balance between multiple invariant activating and inhibitory receptors. The major inhibitory receptors are represented by the killer cell immunoglobulin–like receptor (KIR) family, which represents 17 distinct genes endowed with a high polymorphism, and the CD94/NKG2A heterodimer. Recognizing MHC Class I (MHC-I) molecules on a target cell, inhibitory receptors block NK cell activation (80).

When MHC-I are lost, or their expression is diminished, and this is the case of most tumor developments, NK cells become more susceptible to activation through the involvement of multiple activating receptors such as NKp30, NKp46, NKp44, CD16, NKG2D, DNAX accessory molecule1 (DNAM1), 2B4, and NKp80. Human peripheral blood NK cells can be classified into two subsets in relation to the expression of CD56 and CD16 markers: CD56^dim^CD16^+^ NK cells (comprising 90%–95% of total blood NK cells), characterized by their cytotoxic activity exerted by perforin and granzyme release and mediating antibody-dependent cellular cytotoxicity (ADCC) and CD56^bright^CD16^–^ NK cells (5%–10% of total circulating NK cells), endowed with the capacity of proinflammatory cytokine production, such as IFN-γ and TNF-α and regulatory cytokines like IL-10 (81).

In NSCLC, it has been reported that intratumor NK cells profoundly modify their phenotype and functions, with the expansion of a CD56^bright^CD16^–^ NK cell subset, impairment of cytotoxicity, inhibition of IFN-γ release, and acquisition of pro-angiogenic features (**Table 1**) (82, 83). This tumor-dependent NK cell subset has similarities with a different NK cell subset termed decidual NK (dNK) cells that was identified within decidua. This dNK cell subset was identified as CD56^superbright^CD16^–^ NK cell, and it was shown to be an important regulatory cell in the maternal–fetal interface because of its ability to release not only several pro-angiogenic cytokines and growth factors such as VEGF, PlGF, and CXCL8 but also IFN-γ, which are essential to driving the spiral artery formation during the embryo development (84).

Also, in the context of other types of solid cancers, NK cells accumulating within the tumor microenvironment had immature features and a CD56^bright^CD16^low/−^Perf^low^ phenotype (**Table 1**).

Several soluble factors derived from tumor cells or neighboring innate immune or stromal cells can inhibit and alter NK-cell functions such as TGF-β, PGE2, IDO, adenosine, and IL-10 (85).

We were the first to characterize the decidual-like CD56^bright^CD16^−^ of NSCLC patients with the ability to release pro-angiogenic factors: VEGF, PlGF, and CXCL8 (**Figure 1**) (**Table 1**). The NK-cell subset had the *in vitro* ability to trigger human umbilical vein endothelial cell (HUVEC) migration and the formation of capillary-like structures (86, 87). These peculiar functions are not restricted to the intratumoral NK cells but are also present in their peripheral blood counterpart, suggesting that vascular network induction occurs at a systemic level, too. Moreover, these pro-angiogenic features were detected at a higher intensity in NK cells from patients with squamous cell carcinomas than those with adenocarcinomas. Interestingly, the expansion of pro-angiogenic and decidual-like NK cells was also detected in malignant pleural effusions, colorectal cancer, and prostate cancer patients (88–90).

Recently, Russick et al. analyzed the gene expression profile of intratumoral NK cells and found that in comparison to non-tumorous NK cells, immune cells had a significant decrease of sphingosine-1-phosphate receptor 1 (S1PR1) and CX3CR1 with a concomitant increase of CXCR5 and CXCR6. Intriguingly, they also showed that intratumoral NK cells express inhibitory molecules: CTLA-4 and killer cell lectin like receptor (KLRC1), together with a high expression of CD69 and NKp44, conferring inhibitory capabilities in the context of TIME (91). Indeed, the co-culturing of purified NSCLC NK cells with tumor cells and CD11c^+^ peripheral blood autologous DC in the presence of LPS resulted in the impairment of DC maturation expressed as a percentage of MHC class II and CD86 on DCs. Interestingly, this phenomenon was partially counteracted by the addition of CTLA-4-blocking antibodies. However, the precise mechanism is still not clear, and beyond CTLA-4 expression, other mechanisms could be involved, such as yet-unidentified secreted molecules from NK-cell-derived tumor cells. However, in a tumor mouse model, another possible mechanism of NK-cell-dependent DC inhibition has been identified *via* PD-L1 with PD-1 expressed on DCs (92).

Moreover, a high intratumor density of NK cells is correlated with an improved clinical outcome only in patients with a low infiltration of CD8^+^ T cells, while in patients with elevated CD8^+^ T lymphocyte counts, NK cells conferred a negative impact (91). At later stages, lung tumoral NK cells showed significantly attenuated cytotoxicity, the reduction of levels of granzyme B, perforin, CD107a, IFN-γ, TNF-α, cytotoxic receptor CD27, activating receptor NKG2D, and a higher expression of the inhibitory receptor NKG2A (93).

### Natural Killer T Cells

Natural killer T cells (NKT) cells are a subset of heterogeneous innate-like T lymphocytes CD1d-restricted, recognizing lipid antigens and co-expressing both the T-cell receptor and NK-cell markers, such as CD56, CD16, and NKp46 in humans and NKp46 and NK1.1 in mice. NKT cells can be subdivided into two major subsets: type I and type II NKT cells according to TCR rearrangements and glycolipid reactivity (94, 95).

Type I or invariant NKT (iNKT) cells are cytotoxic cells that express an invariant TCRα chain rearrangement, whereas TCRβ chains present a restricted repertoire. These cells include several subsets called NKT1, NKT2, and NKT17, with similarities to Th1, Th2, and Th17 T-cell subsets, respectively. Type II NKT cells, conversely, display a more diverse repertoire of Vα rearrangements (96, 97). Whereas it is well documented that iNKT cells participate in the anti-tumor response (98), type II NKT cells, on the contrary, enhance tumor growth and metastasis, thus indicating a pro-tumor activity.

The predominant anti-tumor feature of iNKT cells mainly resides in their capacity to release large amounts of Th1 cytokines, such as IFN-γ, in addition to their ability to kill CD1d-positive tumor cells (99, 100) (**Table 1**).

Several studies have shown a relationship between the number and activity of iNKT cells and clinical outcomes, making these cells an interesting therapeutic tool against cancer development and metastasis (99, 101).

However, in NSCLCs, it has been shown that iNKT cells were diminished both in blood and in bronchial lavage samples from patients (102). Moreover, the lung CD1d expression is lowered in NSCLC patients and weak CD1d mRNA expression is significantly associated with poor prognosis. Together, this could indicate a role played by these cells in immunity against NSCLC (102). *In vitro* studies using DNA methyltransferase and histone deacetylase inhibitors on two CD1d-negative NSCLC cell lines: A549 and SK-MES-1, showed the induction of CD1d expression and cytotoxicity directed toward them by iNKT cells, making epigenetic manipulation an interesting immunotherapeutic approach against NSCLC.

A study protocol was mentioned in an ongoing exploring phase I/II clinic trial on 30 patients with EGFR mutation–positive stage III/IV NSCLC that will evaluate the efficacy and safety of using allogeneic CD3^+^CD8^+^ iNKT cells in combination with EGFR-TKIs such as gefitinib (103).

### Dendritic Cells

Dendritic cells (DCs) are antigen-presenting cells (APCs) and consist of three major subsets: myeloid conventional DC1s (cDC1s), myeloid conventional DC2s (cDC2s), and plasmacytoid DCs (pDCs) (104). Several lines of evidence point out that all DC subsets have the capacity to trigger anti-tumor T- cell responses and that DC1s need cooperativity with the other DC subsets (105). Interestingly, it has been shown that DC1s regulate the response to ICIs in mouse models and correlated with better OS in patients with cancer; however, DC1s can be expanded in tumors that resist checkpoint treatment, suggesting that these cells may be altered in their functions (106). Maier et al., using single-cell RNA sequencing in human and mouse NSCLC specimens identified a type of DCs nominated “mature DCs abundant in immunoregulatory factors” (mregDCs), which possessed both immunoregulatory genes (Cd200, Cd274, and Pdcd1lg2) and maturation genes (Cd40, Ccr7, and Il12b). The mregDC function was detected in both DC1 and DC2 subsets upon interaction with tumor antigens and can exert a dual role, both regulatory and immunogenic. It has been shown that the two key steps crucial for regulatory effects driven by mregDCs were the upregulation of PD-L1 and of IL-12, the first was under the control of the receptor tyrosine kinase AXL while the second under the control of IL-4 signaling (106).

Moreover, immature DCs (imDCs), which are present sometimes in high numbers in the tumor microenvironment, can coordinate an immunosuppressive microenvironment together with other innate cells, such as Tregs, MDSCs, and TAMs (**Figure 1** and **Table 1**) (107, 108). Several lung patients could present tertiary lymphoid structures in the stroma of NSCLC, representing a well-organized compartment with lymphocytes and a rise in the density of DC-LAMP^+^ mature DCs, suggesting that these structures might participate in antitumoral immunity. Indeed, several studies showed that these structures were associated with a favorable clinical outcome, together with a Th1 cytotoxic immune response and effective infiltrating CD8^+^ T cells (109, 110).

Interestingly, Inoshima et al. reported an immunohistochemical study in which they analyzed 132 lung cancer specimens showing that a high expression of VEGF and microvessel density is associated with low intratumoral DC infiltration and worse prognosis, whereas low VEGF and high DC are correlated with a better prognosis (111). VEGF that could be produced not only by tumor cells but also by TAMs and NK cells, in addition to having a role in tumor vascular formation, also has a role as an inhibitory molecule for several classes of immune cells, including DCs. Therefore, the subtle regulatory mechanisms involved in the TIME between NK and DC interactions, not yet fully elucidated, as seen above *via* CTLA-4 or PD-1 on NK cells, could underlie the divergent functions of DCs and, in some cases, therefore lead to negative outcomes for the immune response, that is, the expansion of TAMs and Tregs, with a protumoral effect (91, 92).

### Mast Cells

Mast cells are bone marrow–derived immune cells with multiple protective functions against invading microorganisms and harmful agents. These long-lived immune cells exert their regulatory functions in immunity and inflammation by producing key inflammatory mediators, such as tryptase, VEGF, IL-10, TGF-β1, and MMP9, and the relevant data of their anti-tumor or pro-tumor features have been reported (**Figure 1** and **Table 1**). Interestingly, Fontanini et al. investigated the relationship between tumor angiogenesis and survival in 407 NSCLC patients (112). In this study, a worse prognosis was significantly correlated with the increase of the tumor blood vessel network. However, in 2007, a meta-analysis did not confirm an independent prognostic role of vascular density in patients with non-metastatic-treated NSCLC patients (113). The expression of VEGF-A, VEGF-C, and VEGFR-1 was associated with a worse outcome in patients with NSCLC (114). A significant prognostic value of the overexpression of FGF-2 has been reported in patients with operable NSCLC (115). Mast cells are correlated with angiogenesis and a poor outcome in lung adenocarcinoma (116, 117). Angiogenesis assessed by microvessel counts is related with a poor outcome in stage I NSCLC (112, 118–120). Other authors have shown no significant correlations with respect to survival in patients with NSCLC for microvessel density or mast cell infiltration. (121–127). Niczyporuk et al. did not show any correlation between the mast cell count, microvascular count, and survival rate in NSCLC (128). There is no correlation between intratumoral mast cells and angiogenesis in NSCLC (129) and between mast cells and survival in NSCLC (125).

Mast cells present in tumor cell islets are correlated with a marked survival advantage in NSCLC (130). Indeed, whereas mast cell numbers are similar in the tumor stroma of patients with surgically resected NSCLC with no difference to their survival status, there is a substantial survival advantage when mast cells are localized within the clusters of tumor epithelial cells or tumor cell islets (130, 131).

Furthermore, Tomita et al. (132) and Welsh et al. (130) determined a strict correlation between the number of mast cells and a good prognosis in NSCLC. Mast cells have a pro-tumorigenic effect on lung tumor cell lines and an anti-tumorigenic effect *in vivo* (133). Conversely, Stoyanov et al. (133) have reported a significant effect of mast cells and histamine in enhancing NSCLC cell proliferation *in vitro*, whereas in the Lewis lung mouse carcinoma model, they have found that mast cells are crucial negative regulators of cancer development.

### Tregs

Regulatory T lymphocytes (Tregs) are involved in the homeostasis of the immune system, inhibiting autoimmune disorders. Moreover, these cells collaborate with other cells and factors in establishing immunosuppressive TIME (**Figure 1**) (134–136).

The transcription factor forkhead box P3 (FoxP3) is crucial for peripheral naïve T cells to become competent Treg cells (**Table 1**). In lung cancer, Foxp3^+^ Tregs, which suppress auto-reactive T cells to maintain immunological self-tolerance and inhibit autoimmunity, are associated with advanced tumor growth and poor prognosis (137–139). In patients with NSCLC, augmented numbers of blood and intratumoral Tregs are correlated with worse prognosis and a higher risk or recurrence (140).

Several investigations reported significantly higher percentages of CD4^+^CD25^+^FoxP3^+^ Tregs in patients with advanced metastatic NSCLC compared to healthy donors (141–144), whereas the high percentage of CD152^+^CD4^+^CD25^high^FoxP3^+^ Tregs is correlated with a more advanced stage of disease (141, 145). Moreover, two studies reported a prognostic value of blood CD4^+^FoxP3^+^ Tregs in stage I–III NSCLC patients (146, 147).

In NSCLC patients, CD4^+^CD25^+^ Treg subtype functions were associated with their FoxP3, CTLA-4, and IL-7Rα expression, and their blood levels were correlated with the clinical outcome of the patients. Conversely, no difference was found in the percentage of CD4^+^CD25^+^FoxP3^+^ Treg between the entire NSCLC patients and healthy donors (148). Interestingly Tao et al. (139) demonstrated that in NSCLC, there was no significant relationship between the Treg number and the tumor Foxp3 status. However, increased numbers of Tregs were associated with worse overall and relapse-free survival, whereas there was no correlation between the tumor FoxP3 status and survival. In the meantime, when FoxP3^+^ cells were detected within the tumor, the Treg expansion was correlated with the attenuation of worse prognosis. Conversely, the patients in which there was no tumor FoxP3 expression and elevated Treg count had significantly worse overall and relapse-free survival. Collectively, these findings suggest that tumor FoxP3 expression has a better prognostic potential in NSCLC and that, in combination with intratumoral Tregs, the absence of the tumor FoxP3 is correlated with high-risk patients.

## The Lung TIME as a Target for Therapy

The microenvironment of lung cancers is heterogeneous and plays an important role in determining the outcome. The lungs present a unique milieu in which tumors progress in synergy with the TIME, as evidenced by the regions of aberrant angiogenesis, inflammation, and hypoxia. The altered vasculature seen in lung cancers contributes to hypoxia and makes it difficult to efficiently deliver agents through the bloodstream. Hypoxia is associated with an increased risk of metastases as well as resistance to radiation therapy and perhaps chemotherapy. Neutrophils dominate the tumor microenvironment of NSCLC, suppressing T cells and promoting immunosuppression. The multifaceted microenvironment of lung tumors represents many potential targets for the development of novel anticancer agents. As with other cancers, in NSCLC, chronic inflammation represents a major risk factor for the development and progression of cancer.

Tumor-infiltrating CD8^+^ T lymphocytes were associated with improved anti-tumor immunity, as well as with better prognosis in the advanced stage of NSCLC patients (149). Other cell types, such as TAMs and TANs and their subtypes, have their own prognostic effects in NSCLC (150). Furthermore, Tuminello et al. demonstrated the positive role of CD8^+^ T cytotoxic cells, CD20^+^ B cells, and NK cells with survival in patients with early resectable NSCLC (151).

The assessment of tumor inflammation is also of interest, but again, various approaches are being pursued, including a histological assessment of immune cell infiltrates and the mRNA-based expression signatures of immune-related genes. Increased numbers of antitumor CD8^+^ and CD4^+^ T cells have been associated with responding tumors and improved survival, whereas elevated frequencies of Tregs render tumors refractory to immune effector cells (152). The altered vasculature in NSCLC contributes to hypoxia and makes it difficult to efficiently deliver agents through the bloodstream. We have a variety of clinically applicable agents that can modulate the TIME in a way that might improve the response to cytotoxic therapy.

Molecular-targeted therapy represents a fundamental aspect in the treatment of advanced NSCLC. In the past few years, the identification of new molecular subtypes, the search for tumor driver gene mutations, and the development of molecular targeted drugs, such as agents that are able to suppress tumor angiogenesis and regulate tumor immune response, have been the main directions of NSCLC research, clinical diagnosis, and treatment.

In metastatic NSCLC, cytotoxic chemotherapy has been replaced with targeted therapy or immunotherapy. The gene mutation status of EGFR in the tumor tissues of NSCLC is closely related to the efficacy of the TKIs. Getifinib was the first EGFR-TKI tested in patients with advanced NSCLC. The discovery of EGFR mutations provided the biological explanation for the clinical predictors of response to EGFR-TKIs (153). Virtually, all EGFR mutation patients developed acquired resistance to therapy. EMT is implicated in mediating resistance to EGFR inhibitors, chemotherapy, and other targeted drugs in lung cancer (154). In NSCLC, invasive tumor growth is associated with a desmoplastic stroma reaction and the upregulation of EMT markers at the invasive front (155). The inflammatory component of the tumor microenvironment stimulates EMT in lung cancer by contributing to hypoxia, angiogenesis, and the different regulations of miRNAs (156).

Second-generation EGFR TKIs, including afatinib, dacomitinib, and neratinib, have been developed with the intent to delay or overcome acquired resistance (157). Afatinib and dacomitinib resulted in more efficacy than gefitinib in terms of progression-free survival (PFS) and the response rate, whereas gefitinib is associated with fewer side effects (157).

Immunotherapy with anti-PD-1/PD-L1 antibodies has modified the treatment of locally advanced and metastatic NSCLC. The approval of the anti-PD-1 agent pembrolizumab as a standard-of-care first-line treatment in selected patients has made PD-L1 immunohistochemistry a mandatory test in all patients with advanced NSCLC. Immunotherapy alone (pembrolizumab) or in combination with chemotherapy (pembrolizumab or atezolizumab) is the standard of care for first-line therapy in stage IV NSCLC.

In the mouse models of lung cancer, the anti-PD-L1 approach is associated with a rise in exhausted CD8^+^ T lymphocytes (158). At the same time, enhanced numbers of PD-1^+^CD8^+^ T lymphocytes were correlated with reduced survival in stage II and III patients (149). The increased expression of CD38 on T cells after PD-1/PD-L1 ICI favors to acquired resistance by inhibiting CD8^+^ T lymphocyte proliferation and inducing an exhausted phenotype (159). Koh et al. (160) analyzed the correlation between Foxp3^+^ T cells with clinical outcomes before and after anti-PD-1 immunotherapy in patients with advanced NSCLC and found that a higher frequency of blood Tregs 1 week after immunotherapy was associated with prolonged PFS and OS when compared with patients with a low frequency of Tregs. In the meantime, a high expression of TGF-β was correlated with high levels of Tregs and with a favorable clinical outcome.

## Anti-Angiogenic Therapies

Angiogenesis has been strictly related with occurrence, proliferation, and metastasis (161). Targeting the angiogenesis process has been reported to be efficacious in diverse types of cancers, including NSCLC (22). Abnormal vasculature participates in the tumor escape. Anti-angiogenetic agents can normalize blood vessels and thereby reset the TIME from immunosuppressive into immunoreactive. Therefore, combining immunotherapy with anti-angiogenics seems to be a promising strategy for cancer treatments.

The mechanisms appear to be complex and quite a vicious circle where the abnormality of angiogenesis causes an increase in acidity, hypoxia, and interstitial pressure (161, 162), which, later on, are associated with modifications at the molecular and genetic level in blood vessel formation and proliferation, and thus exacerbating and feeding a hostile tumor microenvironment.

In clinical terms, we already have a few monoclonal antibodies approved by the FDA and EMA for the treatment of various cancer types (bevacizumab-binding to VEGF-A, ramucirumab-targeting VEGFR2). By inhibiting the interaction between the VEGF and VEGFR or targeting downstream signaling, these compounds could block tumor angiogenesis. Their efficacy has been proven as a combination therapy with other cytotoxic agents (carboplatin and paclitaxel plus bevacizumab (163), or docetaxel plus ramucirumab (164); meanwhile, as a monotherapy, it showed a limited therapeutic effect in cancer treatment (165).

Ideally, anti-angiogenesis reduces thevascular supply, and thereby impairs tumor cell replication by starving the tumor, but this phenomenon could also decrease the delivery of combination drugs.

Some recent attempts have been taken to solve this paradox. “Vessel normalization” stands at the basis of resetting the perfusion function and structure, enhancing the antitumor immune response by implementing immune cell infiltration (165–168). This procedure gives promises for anti-angiogenesis combined therapies.

Nonetheless, due to the cancer heterogeneity and the multiple aspects of the TIME, the global response rates to ICI therapy are still limited (169). One major factor decreasing the efficacy of ICIs seems to be the elevated recruited numbers of immunosuppressive cells and scarce infiltration of effector cells into the TIME (170).

Latest studies have indicated that pro-angiogenic factors in the tumor microenvironment favor and trigger the development of immunosuppressive cells, and in the meantime, neo vessels impair the infiltration of immune effector cell cancer (171–173).

The use of ICIs in combination with anti-angiogenic agents is hypothesized to be a promising strategy to enhance the global therapeutic efficacy.

There is a progressive and increased understanding on the possible effectivity of anti-angiogenic and IO combination. Nowadays, there are many preclinical and clinical trials suggesting that angiogenesis affects the TIME toward an immunosuppressive state by modifying the recruitment of immune cells (174–178). Later, clinical studies supported that the inhibition of the VEGF/VEGFR signaling can restore the anti-tumor T effector response (172). The use of bevacizumab (avastin) resulted in enhanced cytotoxic T lymphocyte functions in NSCSL as well as in CRC patients (179, 180).

It is well established that TIME is a complex, time-evolving ecosystem consisting not only of tumor cells but also of immune cell blood vessels, stroma cells, and different soluble factors, which turn off antitumor immune responses and favor ineffective immunotherapies (181).

Overstimulation by VEGF signaling in cancer leads to abnormal angiogenesis characterized by increased interstitial fluid pressure, hypoxia, and acidosis. This phenomenon leads to the suppression of the antitumor response through multiple distinct mechanisms (182, 183).

Hypoxia facilitates the infiltration of suppressive immune cells (Tregs, MDSCs, TAMs, and imDCs) by inducing the expression of chemokines (like CSF1, GM-CSF, IL-6, and IL-10) that recruit these immune cells (184); on the other hand, it also inhibits the infiltration of effector T cells through the activation of VEGF (185).

The stimulation and regulation of several key immune cells of TIME such as DCs, MDSCs, Tregs, and TAMs are under the control of VEGF signaling (186, 187). Immunosuppressive factors IL-10, IDO, and TGF-β released by these suppressive immune cells increase even more the immunosuppressive status of TIME (188).

Noteworthy, the inhibition of the VEGF signaling impairs the recruitment of suppressive cells into the tumor microenvironment and, at the same time, increases the infiltration of effector T cells (189). This fact implies that anti-VEGF/VEGFR therapy not only targets the blood vessel function but has the capacity to reactivate antitumor immune responses (173).

In addition to the above negative effects played by VEGF, another effect is related to their capacity to influence an enhanced expression of PD-1, Tim3, and CTLA-4 on activated CD8^+^ T lymphocytes (190). Moreover, VEGF inhibition could result in enhanced IFN-γ production and consequently the induction of PD-L1 expression on tumor cells. This phenomenon provides a strong promise for the anti-angiogenic and ICI drug combined treatment (172, 173).

Currently, we already have the clinical data of a phase III trial Impower 150 (191), which showed a clinical benefit of the combination of IO and bevacizumab plus chemotherapy in NSCLC; in the meantime, other clinical trials are ongoing to assess the safety and efficacy of this new combination therapy in NSCLC (NCT01454102 (CM 012), NCT03689855 (RamAtezo-1), NCT03836066 (TELMA), and others.

## Immunotherapeutic Approaches

Current ICIs directed to CD28-CTLA4/B7 and PD-1/PD-L1 can unleash the power of T cells toward cancer cells by eliminating negative signals that block T-cell functions (192) (193, 194).

Several immune cells such as T cells, NK cells, B cells, and monocytes express PD-1 (195).

Monoclonal antibodies against PD-1, PD-L1, and CTLA-4 are the most used ICIs for NSCLC patients. A number of PD-1, PD-L1, and CTLA-4 inhibitors, including pembrolizumab (196), nivolumab (197), atezolizumab (198), durvalumab (199), avelumab (200), and ipilimumab (201), have been approved for the treatment of advanced NSCLC.

The anti-PD-1 agent pembrolizumab is approved for use as first- and second-line therapy in patients with advanced NSCLC whose tumors express PD-L1 in immunohistochemistry analysis. Nivolumab (anti-PD-1) and atezolizumab (anti-PD-L1) are both indicated for use as second-line therapies regardless of PD-L1 expression. Durvalumab (anti-PD-L1) is approved as a maintenance therapy in patients with unresectable stage III NSCLC whose disease has not progressed following concurrent platinum-based chemoradiotherapy.

Five randomized phase II–III trials testing three ICIs (nivolumab, pembrolizumab, and atezolizumab), all showed a clinically and statistically significant advantage over the same standard comparator docetaxel (21, 22, 197, 202, 203)

ICIs were tested in locoregional NSCLC. A phase III trial demonstrated that adjuvant durvalumab in stage III NSCLC non-progressing after concomitant chemo-radiotherapy improved not only PFS but also OS (204).

Pembrolizumab and nivolumab approval is strictly related with a positive PD-L1 expression.

Checkpoint inhibitors can be used as a combination therapy or as a monotherapy in first- and second-line treatments. The Pacific trial (205) brought immunotherapy in a locally advanced setting and later on, with the publication of IMpower 010 (20), immunotherapy will probably be a practice changing even in early-stage lung cancer.

## Challenges and Future Directions

A prognostic role of many TIME biomarkers is not yet part of the current clinical practice, so further investigations that include larger patient cohorts will be necessary.

ICI alone or in combination with chemotherapy or in combination with other ICIs should be the first-line treatment of choice for patients with advanced NSCLC who do not have contraindications to immunotherapy and whose tumors do not harbor actionable driver mutations. Advances with immunotherapy have offered patients with lung cancer substantial improvements in survival and the quality of life. However, better predictive biomarkers are required to ameliorate the benefit of immunotherapy, and further investigations are needed to find out the mechanisms of resistance to ICIs and how to overcome it. Whereas the PD-1 and PD-L1 ICIs have received accelerated FDA approvals, the development of predictive and prognostic biomarkers for these agents have lagged far behind and remains a crucial area for future research.

The ability to increase the clinical benefit for higher numbers of NSCLC patients and preventing drug resistance will be essential prerequisites to achieve in the near future and related to the acquisition of more knowledge of the induced mechanisms underlying effective antitumor effector responses. The next step will be to better identify patients at the risk of primary or acquired resistance and use increasing amounts of translational research data to develop more effective combination therapies, making the promise of ICIs available to all patients with NSCLC. This is the only way to achieve further advances in cancer immunotherapy and succeed in making the promise of ICIs for all patients.

## Author Contributions

Conceptualization: DB, DR and LM. Text drafting and editing: DB, EC, AI, DR, and LM. Critical revision: DB, EC, AI, and LM. Figure and table preparation: DB. Funds: DB and LM. All authors contributed to the article and approved the submitted version.

## Funding

This study was supported by the Italian Ministry of Health-Grant Giovani Ricercatori 2019 (GR-019-12370076) to DB, and by Fondi di Ateneo per la Ricerca FAR2019 and FAR2020, University of Insubria to LM.

## Conflict of Interest

The authors declare that the research was conducted in the absence of any commercial or financial relationships that could be construed as a potential conflict of interest.

## Publisher’s Note

All claims expressed in this article are solely those of the authors and do not necessarily represent those of their affiliated organizations, or those of the publisher, the editors and the reviewers. Any product that may be evaluated in this article, or claim that may be made by its manufacturer, is not guaranteed or endorsed by the publisher.
